# Henryin, an ent-kaurane Diterpenoid, Inhibits Wnt Signaling through Interference with β-Catenin/TCF4 Interaction in Colorectal Cancer Cells

**DOI:** 10.1371/journal.pone.0068525

**Published:** 2013-07-02

**Authors:** Xingyao Li, Jianxin Pu, Shiyou Jiang, Jia Su, Lingmei Kong, Bingyu Mao, Handong Sun, Yan Li

**Affiliations:** 1 State Key Laboratory of Phytochemistry and Plant Resources in West China, Kunming Institute of Botany, Chinese Academy of Sciences, Kunming, China; 2 State Key Laboratory of Genetic Resources and Evolution, Kunming Institute of Zoology, Chinese Academy of Sciences, Kunming, China; 3 University of the Chinese Academy of Sciences, Beijing, China; University of Kentucky, United States of America

## Abstract

Aberrant Wnt/β-catenin signaling has been strongly associated with the tumorigenesis of human colorectal cancer. Inhibitors of this pathway may then offer therapeutic strategies as well as chemoprevention for this malignant disease. Henryin is an ent-kaurane diterpenoid isolated from *Isodon* *rubescens* var. *lushanensis* , a plant long been used in folk medicine to prevent inflammation and gastrointestinal disease. In the present study, we report that henryin selectively inhibits the proliferation of human colorectal cancer cells with a GI_50_ value in the nano-molar range. Microarray analysis and reporter assays showed that henryin worked as a novel antagonist of Wnt signaling pathway. Henryin reduced the expression of Cyclin D1 and C-myc, and induced G1/S phase arrest in HCT116 cells. Concurrently, henryin did not affect the cytosol-nuclear distribution of soluble β-catenin, but impaired the association of β-catenin/TCF4 transcriptional complex likely through directly blocking the binding of β-catenin to TCF4. We also then analyzed the structure-activity relationship among the ent-kaurane type diterpenoids. Our data suggests that henryin, as a novel inhibitor of Wnt signaling, could be a potential candidate for further preclinical evaluation for colon cancer treatment, and as such warrants further exploration.

## Introduction

Colorectal cancer (CRC) is a common malignancy that, despite advancements in drug treatment and improved diagnosis, remains a major challenge to the medical establishment world-wide. Though the exact pathogenesis of the disease is not clearly understood, chronic inflammation bowel disease is deemed to a risk factor that contributes to the development and metastasis for colorectal cancer [1]. Numerous studies have, however, suggested that aberrant activation of the canonical Wnt/β-catenin signaling pathway play an important role in tumorigenesis. In the absence of Wnt ligands, β-catenin is phosphorylated by a complex including adenomatous polyposis coli (APC)/Dsh/Axin/Gsk3β, which is then ubiquitinated and degraded through the proteasome pathway. When the pathway is activated by the binding of Wnt ligands to its membrane receptor complex, the β-catenin destruction complex dissociates and β-catenin becomes stabilized and enters the nucleus to activate target gene expression in tandem with the TCF transcription factors [2,3].

In colon cancer cells, the Wnt/β-catenin pathway is frequently aberrantly activated [4–6]. According to recent reports from the Cancer Genome Atlas Network, the Wnt pathway is constitutively activated in over 90% of human colorectal cancer by both genetic and epigenetic alterations to a number of genes that are involved in the Wnt signaling pathway, such as β-catenin, APC and Axin2 [7]. Activation of Wnt/β-catenin signaling subsequently increases the expression of Wnt target genes, such as Cyclin D1 [8], C-myc [9] and Survivin [10], all of which are involved in cell proliferation, apoptosis and cell cycle deregulation in the development and progression of malignant colorectal cancer phenotypes [11–13].

To date, advances in the treatment and diagnosis of colorectal cancer are still limited in their effectiveness, prompting us to consider alternative options in moving forward. In folk medicine, some *Isodon* species, widely distributed plants, are used as tea drink to prevent inflammation, gastrointestinal bacterial infections and even tumors. We hypothesized that chemical substances present in some *Isodon* species likely possessed chemopreventive properties as well as chemotherapeutic effects against cancer. In our previous work, many chemical constituents were isolated and characterized from *Isodon* species, but the study of their bioactive profile is very limited and mechanisms are lacking [14–16].

Our present study focuses on screening the compounds that confer anti-tumor effects, especially on human colorectal cancer cells. We found that henryin, an ent-kaurane diterpenoid, isolated from *Isodon* *rubescens* var. *lushanensis* , exhibited selective growth inhibitory effects on human colorectal cancer cells by inhibiting Wnt signaling. Our data showed that henryin interferes with the β-catenin/TCF complex interaction and induces the G1/S phase arrest in colorectal cancer cells. Accordingly, henryin, as novel inhibitor of Wnt/β-catenin pathway, could make a promising drug candidate for both the prevention of colorectal cancer as well as a novel therapeutic.

## Materials and Methods

### Reagents

RPMI 1640 medium, Dulbecco’s modified Eagle medium (DMEM), fetal bovine serum (FBS), antibiotics solution, and Trypsin-EDTA were purchased from HyClone (Logan, UT, USA). The complete protease inhibitor cocktail was purchased from Roche Applied Science (Indianapolis, IN, USA). Mouse monoclonal anti-CyclinD1 and anti-β-catenin antibodies were purchased from BD Biosciences (San Jose, CA, USA). Rabbit monoclonal anti-TCF4 and polyclonal anti-phospho-β-catenin antibody were purchased from Cell Signaling Technology (Danvers, MA, USA). Rabbit monoclonal anti-p21 was purchased from Epitomics, Inc (Burlingame, CA, USA). Mouse monoclonal anti-lamin A/C, anti-β-actin, anti-c-Myc antibodies and other reagents, unless otherwise indicated, were purchased from Sigma-Aldrich (St. Louis, MO, USA). Purified human recombinant proteins β-catenin was purchased from Sino biological inc. Purified human recombinant proteins TCF4 was purchased from OriGene. Dual-luciferase reporter gene assay system was purchased from Promega (Madison, WI, USA). Lipofectamine 2000 was purchased from Invitrogen (Camarillo, CA, USA). The QuantiTect SYBR, Green PCR Kit was obtained from Qiagen (Germany).

### Compounds

Henryin and its analogues were extracted from *Isodon* *rubescens* var. *lushanensis* , as previously described [14–16]. The compounds were dissolved in dimethyl sulfoxide (DMSO).

### Cell lines

Colon cancer cell lines (SW480, HT-29 and HCT116), human lung cancer cell line A549, human normal lung epithelial cell line (Beas-2B) and HEK293T were purchased from ATCC. The normal colonic epithelial cell line (CCD-841-CoN) [17] was kindly gifted by Dr Lin, Li of the Institute of Biochemistry and Cell Biology, Chinese Academy of Sciences (Shanghai, China). Cells were cultured according to the manufacturer’s recommendations. All medium were supplemented with 10% fetal bovine serum (FBS) and 1% antibiotics–antimycotics (100 units/ml penicillin G sodium, 100 mg/ml streptomycin) (Gibco, Invitrogen, USA).

### MTS assay and GI_50_ determination

Cells were seeded in 96-well plates at a density of 5×10^3^cells/well and incubated for 12h in a CO_2_ incubator. Cells were treated with the dose ranging from 0.008 to 4µM of henryin and its analogues, with cisplatin as a positive control for 48h. Then, 20 µl of CellTiter 96® AQueous One Solution Reagent (Promega, Madison, USA) was added and the cells were further incubated at 37 ^°^C for 1-2h. Cell viability was then measured by reading the absorbance at a wavelength of 490 nm. The following formula was used to determine cell growth ratio values: 100* (A490 (sample, T) – A490 (sample, T0)) / (A490 (control, T) – A490 (control, T0)). The GI_50_ values—the concentrations which cause 50% cell growth inhibition—were determined by non-linear regression analysis using TableCurve software.

### Microarray and data analysis

SW480 cells were incubated with 4µM of henryin or dimethyl sulfoxide (DMSO) as a control for 12h. Cells were lysed with TRIzol (Invitrogen, USA) and total RNAs were isolated with the QIAGEN RNeasy Mini Kit (QIAGEN, Germany) before reverse transcription, labeling and hybridization to Agilent Whole Human Genome Oligo Microarray (4×44K) (Agilent Technologies, Palo Alto, CA). The microarray assay was conducted by Shanghai Bio Corporation. After GO annotation and pathway analysis, genes with more than 2 fold alteration in the expression level were selected for further analysis.

### Cell transfection and luciferase reporter assays

For reporter gene assays, cells were seeded in 96-well plates and incubated at 37°C in 5% CO_2_ incubator for 12h. SW480 and HCT116 cells were co-transfected with 100ng of plasmids in total, including 80ng of a well-characterized Wnt/β-catenin pathway responsive firefly luciferase reporter plasmid SuperTOPflash (ST-Luc) and 20ng of pRL-SV40 (Promega) control reporter plasmid using Lipofectamine 2000 (Invitrogen) according to the manufacturer’s instructions. Each well of HEK293T cells was transfected with 200ng of plasmids in total, including 80ng of ST-Luc and 20ng of pRL-SV40 and 10ng of β-catenin or 64ng of wnt1 and empty vector plasmids as indicated. Some 3h after transfection, cells were incubated with various concentrations of compounds for 24h. Cells were lysed with 50µl of Promega lysis buffer in each well and luciferase activity was measured and normalized by pRL-SV40 reporter activity. All experiments were done independently in triplicate. Results are reported as means and standard deviations (SD).

### Reverse transcription and quantitative real-time PCR

Total RNA was extracted from cultured cells with TRIzol and reverse transcription was performed using the SuperScript III Reverse Transcription Kit (Invitrogen) with oligo(dT) priming according to the manufacturer’s instructions. Gene transcripts were quantified by real-time PCR using SYBR Green I and β-actin was used as a control. The primes used were:

Human CyclinD1 forward primer: 5'-AAGTGCGAGGAGGAGGTCTT-3' and the reverse primer: 5'-GGATGGAGTTGTCGGTGTAGA-3'.

Human C-myc forward primer: 5'-CTTCTCTCCGTCCTCGGATTCT-3' and the reverse primer: 5'-GAAGGTGATCCAGACTCTGACCTT-3'.

Human β-actin forward primer: 5'-CGCGAGAAGATGACCCAGAT-3' and the reverse primer: 5'-GATAGCACAGCCTGGATAGCAAC-3'.

RT-PCR experiments were performed in triplicates and PCR efficiency with given primers was between 95 and 100%. Melting curves were also performed for monitoring the primer-specific amplifications.

### Cytosol and nucleus fractionation and western blotting analysis

SW480 cells were treated with the henryin in 6-well plates. Fractionated nuclear and cytosolic lysates were obtained using nuclear extraction buffer and hypotonic lysis buffer, respectively. All protein extraction buffers were supplemented with Complete protease inhibitor cocktail and phosphatase inhibitor cocktail (Roche). The protein concentration of each crude lysate was determined using the Enhanced BCA Protein Assay Kit (Beyotime, Shanghai, China). The normalized amounts of the lysate samples were loaded on SDS-PAGE and transferred to the polyvinylidene difluoride (PVDF) membranes (Millipore, Bedford, MA, USA). Immunoblotting was performed using antibodies detecting phospho-β-catenin (Ser33/37/Thr41), β-catenin, Axin2, CyclinD1, C-myc, Survivin, P21, TCF4, with Lamin A/C and β-actin used as loading controls. HRP conjugated anti-mouse IgG and anti-rabbit IgG were used as secondary antibodies. SuperSignal West Pico Chemiluminescent Substrate (Thermo scientific) was used to detect chemiluminescence, and blots were imaged using the Luminescent Image Analyzer LAS-4000mini System (GE, USA).

### Co-immunoprecipitation analysis

SW480 cells were cultured in 6-well plates, treated with henryin and its analogs enmenol and minheryin C for 12h, lysed in protein lysis buffer (50 mM Tris-HCl, pH 7.4, 150mM NaCl, 1% Triton X-100, 1mM EDTA, and proteinase inhibitors) and centrifuged at 14000×g for 15 min at 4°C. The supernatant was incubated with a specific primary antibody overnight at 4°C and then incubated with A/G PLUS agarose (Santa Cruz Biotechnology, Inc.) for at least 2h. Beads were then washed five times and re-suspended in 50 µl SDS loading buffer. Western blot analysis was performed on the samples.

### *In vitro* binding assay

To assay the effect of compounds in inhibiting β-catenin/TCF4 binding, purified recombinant proteins β-catenin (0.8µg) and TCF4 (0.5µg) were incubated with henryin and its analogs enmenol and minheryin C at indicated concentrations at 4°C for 2h, respectively. β-catenin/TCF4 complexes were pulled down using protein A/G PLUS agarose beads saturated with β-catenin antibody. Detection was then performed using TCF4 antibody in western blotting analysis.

### Cell cycle analysis

HCT116 cells (5×10^5^cells/well in 12-well plates) were incubated with henryin for 0h, 12h and 24h, respectively. All adherent cells were collected and washed twice with PBS. Cells were fixed with 70% ethanol overnight. Fixed cells were washed with PBS, and then stained with a 50µg/ml propidium iodide (PI) solution containing 50µg/ml RNase A for 30 min at room temperature. Fluorescence intensity was analyzed by FACSCalibur1 flow cytometer (BD Biosciences, San Jose, CA, USA). The percentages of the cells distributed in different phases of the cell cycle were determined using ModFIT LT 2.0.

### Statistics

Data are presented as the mean ± SD for the indicated numbers of independently performed experiments. Non-linear regression analysis for the calculation of GI_50_ or IC_50_ values was performed by TableCurve. Statistical significance was analyzed by Student’s *t*-test or one-way ANOVA in SigmaStat 3.1. Differences were considered to be statistically significant when P < 0.05.

## Results

### Henryin selectively inhibits the growth of colorectal cancer cells and induces G1 phase arrest of the cell cycle

**Figure 1:**
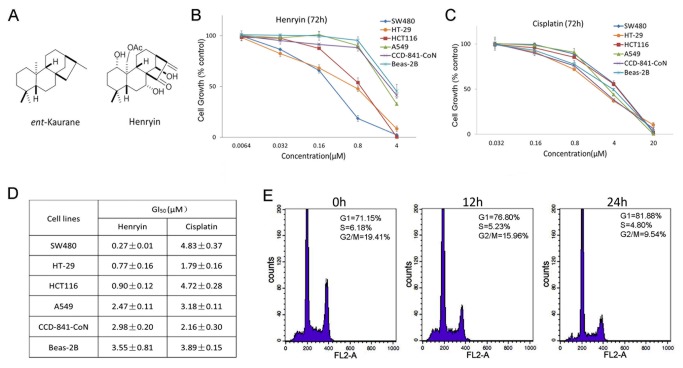
Henryin selectively inhibits the growth of colon cancer cells and induces G1 phase arrest. (A) The structures of henryin and ent-kaurane are shown. (B) Growth inhibition of henryin on various cell lines, including colorectal cancer cell lines (SW480, HCT116 and HT29), normal colonic epithelial cell line CCD-CoN-841, lung cancer cell line A549, and normal lung epithelial cell line Beas-2B, determined by MTS assays with cisplatin used as a control (C). Cells were treated with henryin up to 72h at various concentrations. The absorbance was measured at 490 nm. All sampling was done in triplicates and the data are presented as mean ± SD. (D) The median growth inhibition concentration (GI_50,_ 72h) values of henryin and cisplatin for various cell lines. (E) Representative histograms depicting cell-cycle distribution as analyzed by flow cytometry in HCT116 cells treated with 4µM of henryin for 0h, 12h and 24 h respectively. Counts of G1 phase cells increased remarkably in the treated cells in a time-dependent manner.

The growth inhibition effects of henryin on human cancer cells were primarily evaluated with MTS assays. As shown in Figure 1B, henryin exhibited stronger growth inhibitory effects on the colon cancer cells (SW480, HT-29 and HCT116) than any other types of cancerous (lung cancer cell line A549) or normal cells (human normal colonic epithelial cell line CCD-841-CoN and normal lung epithelial cell line Beas-2B). As a control, cisplatin had similar inhibitory effects among all the different cell lines studied (Figure 1C). Analysis of the GI_50_ values of henryin and cisplatin on the different cell lines also supported the observation that henryin had a selective inhibitory effect on colon cancer cells (Figure 1D). The growth inhibitory effects of henryin on SW480 cells showed a time-dose dependent manner (data not shown). Cell cycle analysis showed that the cells were arrested at the G1 phase in HCT116 cells treated with henryin in a time-dependent manner (Figure 1E). These results together suggest that henryin exhibited selective growth inhibition on colorectal cancer cells.

### Henryin interferes with Wnt signaling in CRC cells

**Figure 2:**
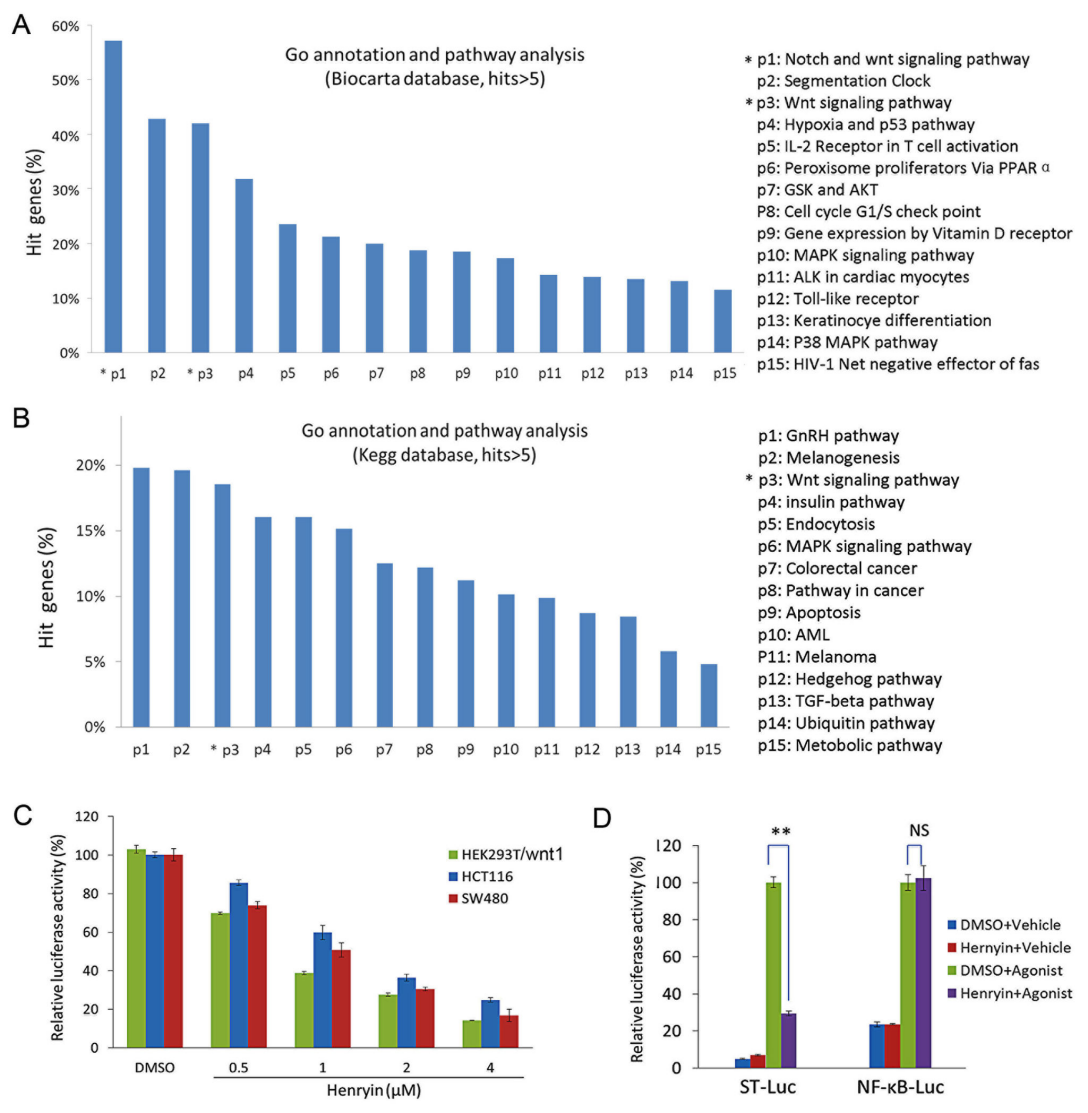
Henryin inhibits Wnt/β-catenin signaling. (A) (B) Microarray assay and data analysis of differential gene expression under henryin treatment. SW480 cells were treated for 12h with 4µM of henryin with 0.5% DMSO used as a control in the assay. GO annotation and pathway analysis were employed from the BioCarta and KEGG databases, respectively. The genes with fold alteration of at least >2 (up-regulated) or <0.5(down-regulated) were taken as differentially expressed genes. Data analysis and the statistics were generated only when the hits > 5 in each signaling pathway. Representative pathways obtained after microarray data analysis are shown. (C) Henryin inhibits the Wnt reporter expression in a dose-dependent manner in wnt1 transfected HEK293T cells, and in colorectal cancer cells, SW480 and HCT116. Three hours after transfection of the Wnt1 and/or ST-Luc, DMSO or henryin with indicated dosage was added to the cells for additional 24h and luciferase activity was then measured. (D) Henryin (4µM) preferentially inhibits the Wnt signaling (ST-Luc) over the NF-κB signaling pathway (NF-κB-Luc) in HEK293T cells. Wnt signaling was stimulated by transfection of wnt1 and NF-κB signaling was stimulated with 25ng/mL TNFα. Each bar is the mean ± SD from three independent experiments. *P<0.05, **P<0.01, relative to vehicle control. NS, not significant.

In order to investigate the underlying mechanisms through which henryin inhibits the growth of colorectal cancer cells, we carried out a microarray analysis and alterations of gene expression were assayed in henryin treated SW480 cells. The genes were filtered by setting criterion of fold alteration >2 (up-regulated) or <0.5 (down-regulated) as differentially expressed genes. Both GO annotation and pathway analysis showed that henryin strongly affects the Wnt signaling pathway as well as other altered pathways that are associated with colorectal cancer biological processes in both the KEGG and BioCarta databases (Figure 2A-B).

Following the microarray analysis, we checked the effect of henryin on Wnt/β-catenin signaling using the TOPflash reporter assay. In wnt1 transfected HEK293T cells, henryin inhibited the Wnt signaling reporter expression in a dose-dependent manner after 24h of treatment (Figure 2C). Wnt signaling is constitutively activated in SW480 and HCT116 cells due to APC truncation and β-catenin mutation, respectively. In both cell lines, the Wnt signaling was also inhibited by henryin dose-dependently (Figure 2C). Concurrently, henryin showed no clear effect on the activity of NF-κB signaling responsive luciferase reporter (NF-κB-Luc) (Figure 2D). This data suggests that henryin specifically inhibits Wnt/β-catenin signaling.

**Figure 3:**
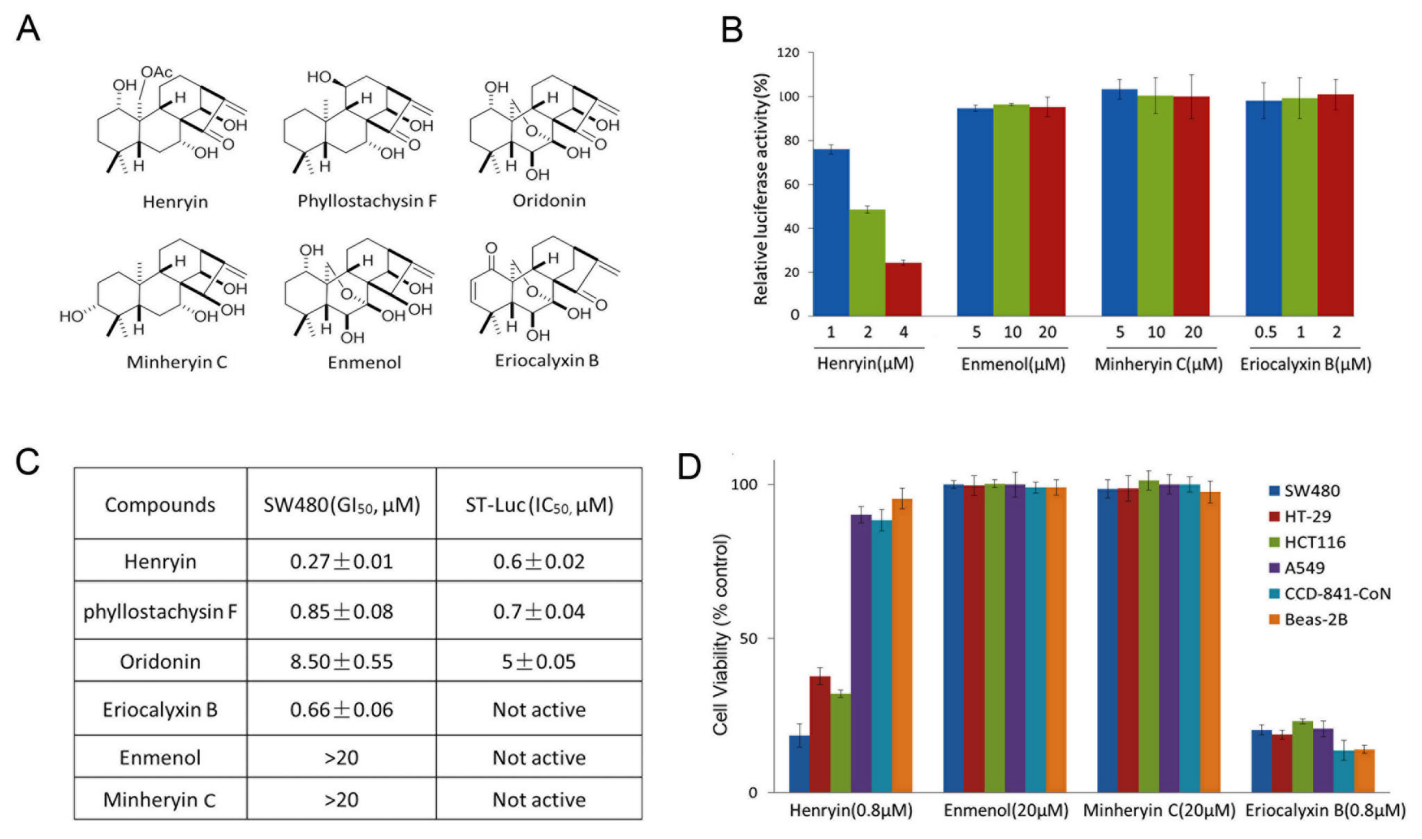
Structure-activity relationship of Henryin. (A) Structures of henryin and its analogues. (B) Effects of henryin analogues on Topflash activity in HEK293T cells. Cells were transfected with Wnt1 and ST-Luc for 3h and then incubated with henryin and its analogues of different dosages for 24h and afterward luciferase activities were measured. (C) The median growth inhibition (GI_50,_ 72h) and ST-Luc inhibition (IC_50,_ 24h) values of henryin and its analogues in SW480 cells are shown. (D) Effects of henryin analogues on the growth of various cell lines (48hours). Data shown is the mean ± SD from three independent experiments.

To characterize the structure-activity relationship of henryin, we investigated the activities of additional henryin analogues (Figure 3A) and examined their effects on Wnt signaling with ST-Luc reporter assay. Two of the compounds, phyllostachysin F and oridonin, which possess a ketone group at C-15 and 14β-OH, similar to henryin, also exhibited distinct inhibitory effects on the ST-Luc activity. Meanwhile, the henryin analogues enmenol, minheryin and eriocalyxin B, which lack the ketone group at C-15 or 14β-OH, showed no effect on the Wnt reporter activity (Figure 3B-C). Subsequently, we further examined the growth inhibition effects of enmenol, minheryin and eriocalyxin B. Enmenol and minheryin C, consistent with their non-inhibitory activities on Wnt signaling, exhibited no growth-inhibitory effect on the colorectal cancer cells (Figure 3D). Notably, eriocalyxin B showed strong cytotoxicity against colorectal cancer cells, lung cancer cells and even the normal cell lines (Figure 3D). This broad spectrum cytotoxicity of eriocalyxin B could be due to its inhibitory activity on the NF-κB pathway, as a reported NF-κB inhibitor [18]. These data suggest that the ketone group at C-15 and 14β-OH are responsible for henryin’s inhibitory effect on Wnt signaling.

### Henryin inhibits endogenous Wnt target gene expression

**Figure 4:**
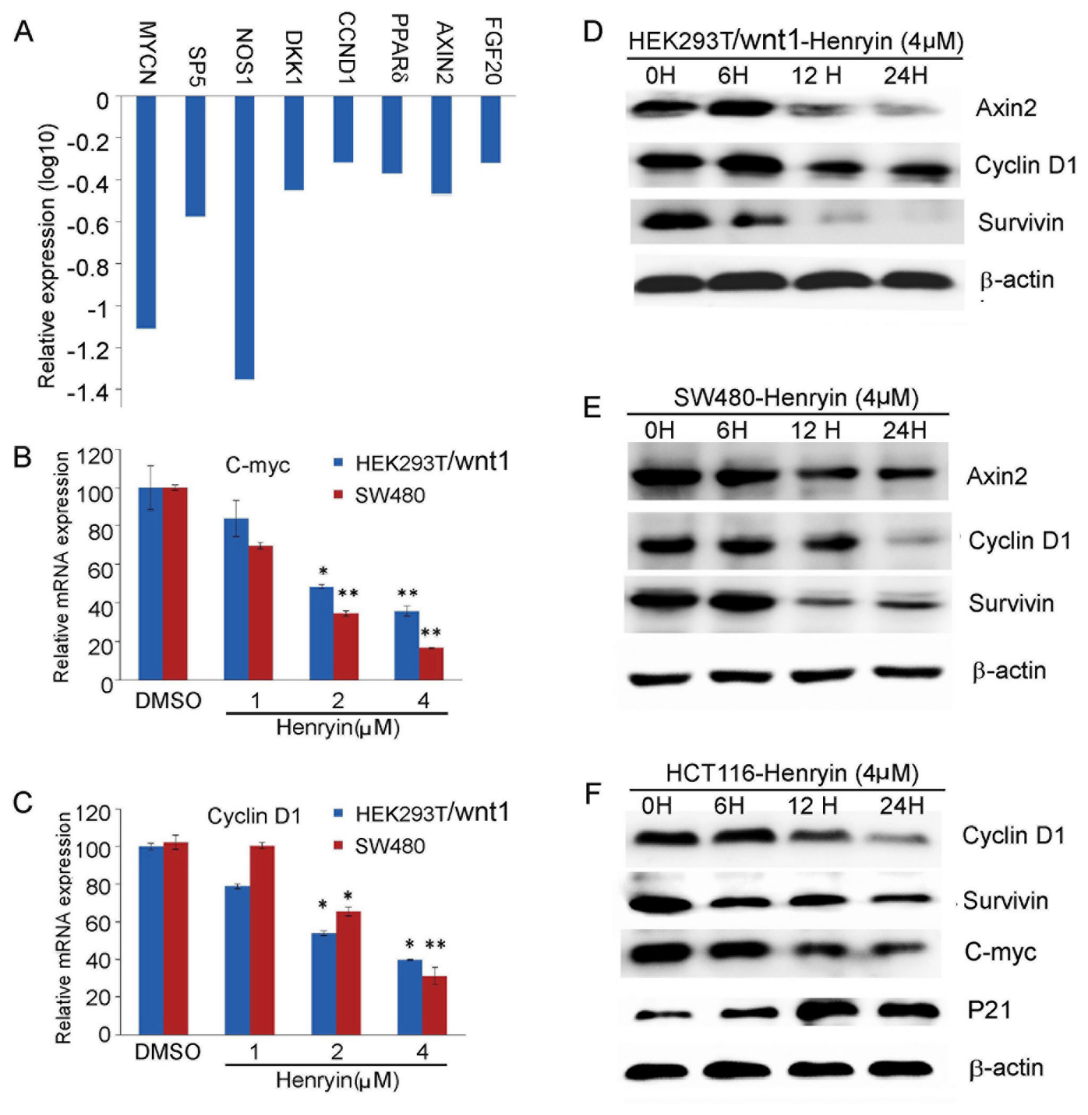
Henryin inhibits endogenous Wnt target gene expression. (A) Henryin down-regulates the expression of Wnt target genes in microarray assay. (B–C) Henryin inhibits Cyclin D1 and C-myc expression in wnt1 transfected HEK293T cells and SW480 cells. The cells were treated with indicated dosages of henryin for 12hours. The levels of Cyclin D1 and C-myc mRNAs were determined by real-time PCR and normalized to β-actin. (D) The effects of henryin on the expression of Axin2, CyclinD1 and Survivin proteins in wnt1 transfected HEK293T cells. The cells were transfected with wnt1 3 hours before henryin treatment. (E) The effects of henryin on the expression of Axin2, CyclinD1 and Survivin proteins in SW480 cells. (F) The effects of henryin on the expression of CyclinD1, Survivin, C-myc and P21 proteins in HCT116 cells. The cells were treated with henryin for 6, 12, 24h and cell extracts were assayed with western blotting of the indicated proteins. β-actin was used as a loading control. *P<0.05, **P<0.01, relative to vehicle control.

We checked the expression of endogenous Wnt target genes in our microarray data and found that the expression of Wnt target genes, including Axin2, Cyclin D1, SP5, DKK1, NOS1, PPARδ and FGF20, were significantly down-regulated after treatment with henryin (Figure 4A). To confirm the inhibitory effects of henryin on the expression of Wnt signaling target genes, we treated wnt1 transfected HEK293T and SW480 cells with henryin for 12h and monitored the expression of C-myc and Cyclin D1 by RT-PCR. The results showed that henryin reduced their expression in a dose-dependent manner (Figure 4B-C). We also employed western blot analyses to detect the protein expressions of Cyclin D1, Survivin, Axin2 and C-myc in HEK293T, SW480 cells and HCT116 cells treated with henryin. Consistent with the above results, the protein level of Axin2, Cyclin D1, C-myc and Survivin were reduced by henryin within 24h of treatment (Figure 4D–F).

### Henryin interferes with the association of β-catenin/TCF complex

**Figure 5:**
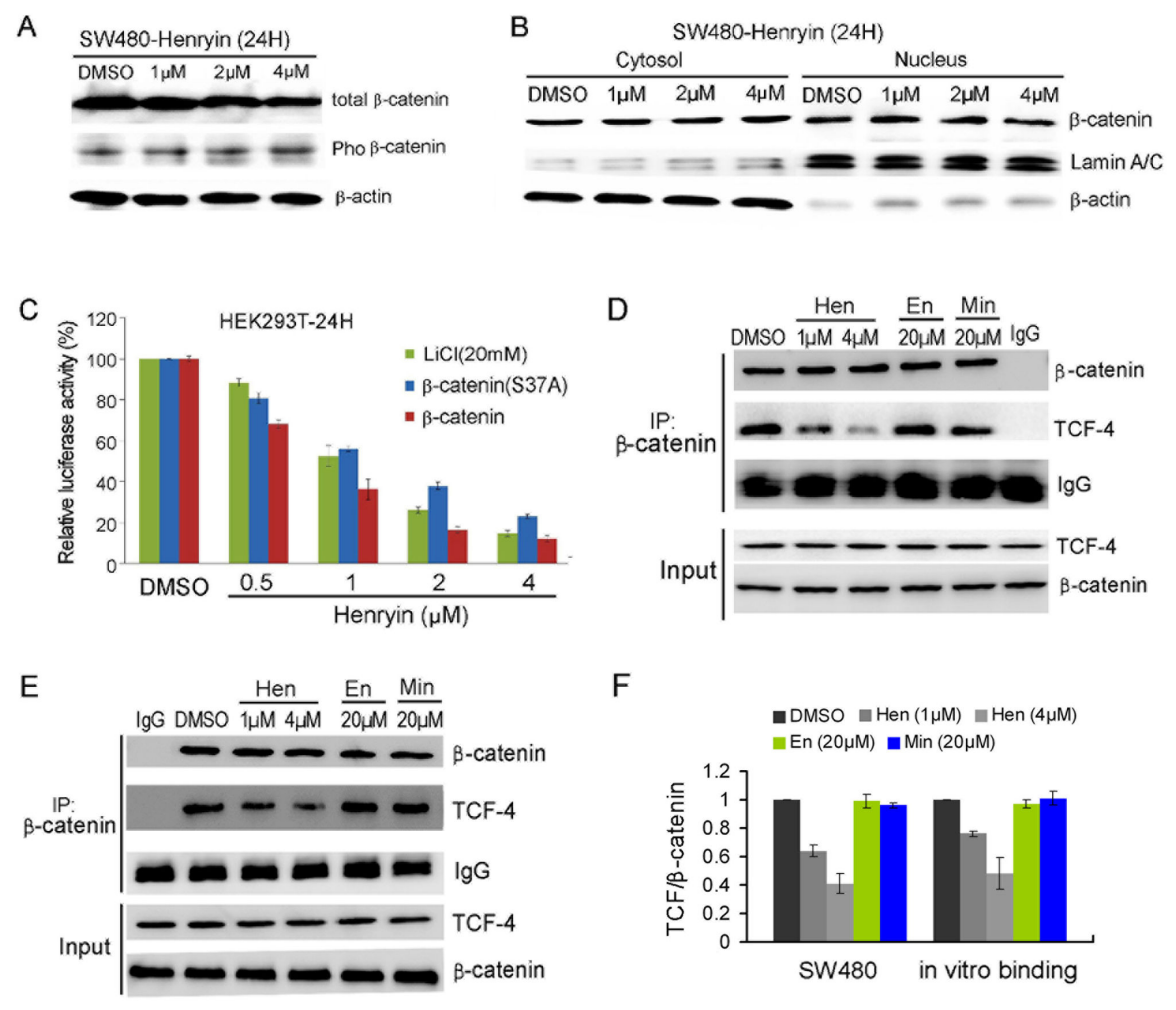
Henryin interferes with the association of β-catenin with TCF4. (A) Henryin does not affect the amount of total β-catenin and pho-β-catenin in SW480 cells. (B) Henryin does not affect the distribution of β-catenin in cytosol and nucleus fractions in SW480 cells. SW480 cells were treated with the indicated dosages of henryin for 24h. The cell extracts or fractions were prepared and analyzed by western blotting. Lamin A/C and β-actin were used as loading controls of nuclei and cytoplasm proteins, respectively. (C) Henryin antagonizes the Wnt signaling stimulated with LiCl or β-catenin. HEK293T cells were transiently transfected with ST-Luc and β-catenin or constitutively active β-catenin (S37A) plasmids, respectively, or treated with LiCl (20mM). Around 3h after transfection or treatment, henryin was added and cells were incubated for an additional 24h. Each bar is the mean ± SD from three independent experiments. (D) Henryin, but not enmenol and minheryin C, reduced the TCF4 levels associated with β-catenin in SW480 cells in a dose dependent manner. Cells were treated with the indicated dosages of henryin and its analogs enmenol and minheryin C for 12h and immune-precipitation was performed by β-catenin antibody with mouse IgG as a control, and then TCF4 was detected by western blotting. (E) Henryin directly disrupts the interaction of β-catenin with TCF4 in the in vitro binding assay. Human recombinant β-catenin (0.8µg) and TCF4 (0.5µg) were mixed, incubated with different concentrations of henryin and its analogs enmenol and minheryin C, and the mixture was subjected to co-immunoprecipitation with β-catenin antibody and western blotting analysis with TCF4 antibody, with mouse IgG used as control. (F) Quantification of the western blots shown in D and E. The software ImageJ was used to analyze the intensities of bands. Data was presented as mean ± SD from three experiments. Hen, henryin, En, enmenol, Min, minheryin C.

One hallmark of activation of Wnt signaling is the stabilization and nuclear accumulation of β-catenin in colorectal cancer cells. We checked the effect of henryin on the level and distribution of β-catenin in SW480 cells. SW480 cells were treated for 24hours with henryin at various concentrations and total β-catenin and phosphorylated form of β-catenin were examined (Figure 5A). Meanwhile, the nuclear and cytoplasmic β-catenin were also examined using western blot analysis. The results revealed that henryin treatment affects neither the level of total and phosphorylated form of β-catenin, nor the distribution of β-catenin in the nuclear and cytoplasmic compartments (Figure 5B), suggesting that henryin targets the Wnt pathway downstream of β-catenin. Consistent with the above result, the activity of ST-Luc in the HEK293T cells with β-catenin overexpressed was significantly reduced by henryin, as well as the stabilized form of β-catenin due to the S37A mutation (Figure 5C). LiCl is an inhibitor of GSK-3β that blocks the phosphorylation and subsequent degradation of β-catenin, thereby resulting in the stabilization of β-catenin [19]. Unsurprisingly, henryin antagonized LiCl-induced activation of the canonical Wnt signaling as well (Figure 5C). Taken together, these data suggest that henryin does not affect the stabilization of β-catenin or its nuclear translocation, but rather targets the Wnt signaling downstream of it.

In the nucleus, β-catenin needs to bind transcription factors of the TCF family to regulate target gene expression. We checked whether henryin impairs the β-catenin/TCF interaction. SW480 cells treated with henryin were immune-precipitated by β-catenin antibody or mouse IgG as a control, and TCF4 was detected by western blotting and the blots were analyzed with ImageJ for intensities of the bands. The results showed that henryin strongly inhibited the binding of TCF4 to β-catenin in a dose-dependent manner in SW480 cells but not its analogs enmenol and minheryin C (Figure 5D, 5F), which is consistent with the inhibitory activities on the Wnt signaling and the cancer cell growth of the compounds.

In order to determine whether henryin can directly disrupt the interaction of β-catenin with TCF4, in vitro binding assays were performed with puriﬁed recombinant β-catenin and TCF4 proteins. The result showed that henryin strongly inhibited the binding of TCF4 to β-catenin in the in vitro binding assays, whereas its analogs enmenol and minheryin C exhibited no any effect as expected (Figure 5E, 5F).

## Discussion

Targeting Wnt signaling may represent a promising strategy to eradicate malignant disease with abnormal activation of this pathway. Accordingly, identifying new inhibitors of the Wnt/β-catenin signaling pathway are of great importance and significance. This inhibition may be achieved by disrupting the interaction of β-catenin/TCF [20] or CREB binding protein (CBP) [21]. Stabilizing Axin2 [22,23] and activation of CK1α also were taken as effective mechanisms to inhibit Wnt signaling for targeted therapeutics against colon cancer [24]. In previous research, several compounds have been found to possess the ability to interrupt β-catenin/TCF association directly, such as PKF115-854 and CGP049090, and the like.

In recent years, there has been significant interest in the search for Wnt/β-catenin signaling antagonists from natural products [25]. Indeed, many structure types of natural products have been identified that inhibit Wnt/β-catenin signaling, including the Murrayafoline A [26], Tetrandrine alkaloid [27], curcumin [28], EGCG [29,30], ﬂavonoids [31,32], Magnolol [33] and others [34,35]. Recently, resveratrol was reported as being capable of inhibiting Wnt signaling downstream of β-catenin, thereby contributing to the repression of colon cancer tumorigenesis [36].

Henryin, an ent-kaurane diterpenoid, isolated from *Isodon* *rubescens* var. *lushanensis* preferentially induced colorectal cancer cells death while leaving normal colonic CCD-CoN-841 and normal lung epithelial Beas-2B cells less affected (Figure 1B, D). We identified the signaling pathways affected by henryin in colon cancer cells by microarray assay, among which the Wnt signaling pathway was found to be the strongly altered one. Consistent with the microarray data, in both wnt1 transfected HEK293T cells and colorectal cancer cells, henryin inhibited Wnt-responsive reporter activity in a dose-dependent manner (Figure 2C). Henryin decreased the expression of endogenous Wnt target genes (Figure 4A–F) and interfered with the association of β-catenin/TCF transcription complex likely by directly blocking the binding o f β-catenin to TCF 4 (Figure 5D, 5E, 5F). Taken together, these data suggested that the anti-proliferation activity of henryin in colorectal cancer cells was associated with its inhibition of Wnt signaling. Critically, henryin specifically inhibits Wnt/β-catenin signaling but not NF-κB signaling (Figure 2D), while eriocalyxin B, one of its analogues, suppresses NF-κB signaling but not Wnt/β-catenin signaling. This difference in inhibition explains why henryin preferentially induced colorectal cancer cells death but eriocalyxin B generally acted as a cytotoxic agent (Figure 3D).

As a key target gene of Wnt signaling, the oncogene C-myc contributes to increased cell proliferation in a variety of human cancers. Recent insights into its expression and function have led to therapeutic opportunities [37]. Cyclin D1 is a known cell cycle protein that is frequently over-expressed in human colon cancer and plays a prominent role in driving tumorigenesis. In henryin treated cells, the expression levels of both Cyclin D1 and c-Myc were reduced. At the same time, the expression of P21, another cell cycle related gene which is negatively regulated by C-myc, increased (Figure 4B–F). All the above data are in agreement with our flow cytometric cell cycle analysis wherein henryin attenuated G1 phase of the cell cycle and inhibited the growth of HCT116 cells (Figure 1E).

In conclusion, henryin, an ent-kaurane diterpenoid isolated from *Isodon* species, preferentially induced colorectal cancer cell death. In the present study, we proposed and illustrated the molecular mechanisms modulated by henryin responsible for anti-tumor proliferation effects. *Isodon* species have long been used in folk medicine. Our findings that henryin is a novel inhibitor of Wnt/β-catenin signaling will provoke its use as a potential anticancer agent. Obviously further studies on the in vivo efficacy as well as the pharmacodynamic effects of henryin will provide potentially novel therapeutic strategies for colorectal cancer with minimum adverse effects on normal tissues.
